# Unlocking the adaptive advantage: correlation and machine learning classification to identify optimal online adaptive stereotactic partial breast candidates

**DOI:** 10.1088/1361-6560/ad4a1c

**Published:** 2024-05-30

**Authors:** Joel A Pogue, Joseph Harms, Carlos E Cardenas, Xenia Ray, Natalie Viscariello, Richard A Popple, Dennis N Stanley, D Hunter Boggs

**Affiliations:** 1 Department of Radiation Oncology, University of Alabama at Birmingham, Birmingham, AL, United States of America; 2 Department of Radiation Medicine and Applied Sciences, University of California San Diego, San Diego, CA, United States of America

**Keywords:** adaptive radiotherapy, early-stage breast cancer, machine learning, accelerated partial breast irradiation, CBCT-based adaption, plan comparison

## Abstract

**Objective.:**

Online adaptive radiotherapy (OART) is a promising technique for delivering stereotactic accelerated partial breast irradiation (APBI), as lumpectomy cavities vary in location and size between simulation and treatment. However, OART is resource-intensive, increasing planning and treatment times and decreasing machine throughput compared to the standard of care (SOC). Thus, it is pertinent to identify high-yield OART candidates to best allocate resources.

**Approach.:**

Reference plans (plans based on simulation anatomy), SOC plans (reference plans recalculated onto daily anatomy), and daily adaptive plans were analyzed for 31 sequential APBI targets, resulting in the analysis of 333 treatment plans. Spearman correlations between 22 reference plan metrics and 10 adaptive benefits, defined as the difference between mean SOC and delivered metrics, were analyzed to select a univariate predictor of OART benefit. A multivariate logistic regression model was then trained to stratify high- and low-benefit candidates.

**Main results.:**

Adaptively delivered plans showed dosimetric benefit as compared to SOC plans for most plan metrics, although the degree of adaptive benefit varied per patient. The univariate model showed high likelihood for dosimetric adaptive benefit when the reference plan ipsilateral breast V15Gy exceeds 23.5%. Recursive feature elimination identified 5 metrics that predict high-dosimetric-benefit adaptive patients. Using leave-one-out cross validation, the univariate and multivariate models classified targets with 74.2% and 83.9% accuracy, resulting in improvement in per-fraction adaptive benefit between targets identified as high- and low-yield for 7/10 and 8/10 plan metrics, respectively.

**Significance.:**

This retrospective, exploratory study demonstrated that dosimetric benefit can be predicted using only ipsilateral breast V15Gy on the reference treatment plan, allowing for a simple, interpretable model. Using multivariate logistic regression for adaptive benefit prediction led to increased accuracy at the cost of a more complicated model. This work presents a methodology for clinics wishing to triage OART resource allocation.

## 1. Introduction

Accelerated partial breast irradiation (APBI) utilizes localized radiation therapy (RT) for treating early-stage breast cancer by exclusively treating the lumpectomy cavity and surrounding tissue, as opposed to the whole breast (Smith *et al* 2009), since recurrence typically occurs within the same quadrant and microscopic malignancy is usually confined within 1 cm of the surgical resection margin (Fisher *et al* 1992, Vicini *et al* 2004). APBI confers similar oncologic outcomes and improved cosmesis compared to whole breast RT with appropriate patient selection (Strnad *et al* 2016), and enables treatment delivery in less than two weeks rather than 3–6.5 weeks. Historically, intra-operative radiotherapy (IORT) (Vaidya *et al* 2005), high dose rate brachytherapy (HDR) (Polgar *et al* 2013, Strnad *et al* 2016), and linear-accelerator (linac) based 3D conformal radiotherapy (Baglan *et al* 2003, Vicini *et al* 2003) or intensity modulated radiation therapy (IMRT) (Livi *et al* 2015) have been the preferred APBI delivery techniques. HDR and IORT offer excellent localization due to direct applicator insertion into the lumpectomy cavity, but these techniques are more invasive than linac-based methods which utilize external localization. The use of stereotactic body radiation therapy (SBRT) for APBI has more recently garnered popularity due to reduced planning target volume (PTV) size, correlating with lower rates of fat necrosis in patients receiving five fraction SBRT (Rahimi *et al* 2017, 2021, Liu *et al* 2022).

However, the seroma cavity can vary in both volume and location between the post-surgery computed tomography (CT) simulation and the first treatment because the treatment planning process for linac-based APBI can take weeks, with volumetric decreases of the post-operative surgical bed as high as 50% (Jeon *et al* 2017, Sager *et al* 2018). Additionally, breast tissue is pendulous, presenting unique patient-positioning challenges due to additional set-up uncertainty. For these reasons, APBI often requires adaptive radiotherapy where the treatment plan is re-optimized for alterations in lumpectomy cavity sizes and anatomical breast variations. Offline adaptive RT typically results in only one or two re-plans per treatment course (Yan *et al* 1997, Sonke *et al* 2019), while online adaptive radiotherapy (OART) enables daily, real-time replanning via onboard imaging and advanced computing. As such, OART allows for the maximal reduction of normal tissue dose (Foroudi *et al* 2011, Moazzezi *et al* 2021, Astrom *et al* 2022, Mao *et al* 2022), especially in stereotactic treatment regimens (Henke *et al* 2019, Schiff *et al* 2022). Our institution has demonstrated improved target coverage, OAR sparing, and plan quality with online adaption of APBI (Pogue *et al* 2023b); although, prospective clinical trials are necessary to verify a reduction in toxicity, and other studies showed only improved target dose without increased OAR sparing (Montalvo *et al* 2023).

However, despite the clinical potential of OART, there are many challenges which prevent widespread clinical adoption, including uncertainties in dose calculations from on-board imagining and synthetic CT generation, increased time required for autocontouring and plan optimization/calculation, inability to perform traditional patient-specific quality assurance, and significantly increased resource allocation compared to the standard of care (SOC) (Bertholet *et al* 2020). To account for anatomic variation when optimizing per-fraction plans, more time is required from the physician and physicist during the initial planning process for reviewing treatment intents and derived structures (Schiff *et al* 2023). At treatment, OART requires an expert trained in organ delineation to evaluate and/or edit all auto-contoured normal tissue and targets that affect plan optimization and evaluation. Importantly, these contours are often edited by a medical physicist whose presence, under non-adaptive SOC, is not required at the machine (Branco *et al* 2023), though some clinics have trained radiation therapists to contour (Shepherd *et al* 2021). These contours then require careful and timely offline review by a physician substantially increasing the time they spend reviewing daily plans compared to SOC IGRT. Due to the increased risks and challenges involved, many institutions require physician and physics presence for the entire adaptive process, further increasing the associated treatment cost. Due to the extra processes and safety checks, OART treatment times are substantially longer (e.g. 25 min for cervical cancer treatments (Yock *et al* 2021) and 31 min for stereotactic APBI treatments (Pogue *et al* 2023b)), thus reducing patient throughput or extending the overall treatment day, which has subsequent impacts on hospital costs and staffing needs.

As a result of this increased staff and patient burden, identification of patients that would receive the most benefit from OART is crucial. It has been hypothesized that a priori prediction of prostate cancer patients receiving large adaptive benefit would mitigate resource costs associated with OART (Moazzezi *et al* 2021), and Ghimire *et al* performed multivariate analysis to forecast per-patient OART dosimetric benefit for cervical cancer patients (2023). Additionally, Yock *et al* demonstrated the feasibility of per-metric adaptive triggers for standard and hypo-fractionated pelvic treatments (2023). However, although it has been shown that minimizing the PTV to breast volume ratio reduces toxicity resulting from non-adaptive APBI (Rahimi *et al* 2017), no investigation identifying optimal OART APBI candidates has been performed to the authors’ knowledge. The primary aim of this study is to develop predictive models to triage patients such that the patients most likely to receive dosimetric benefits from OART are allocated to adaptive pipelines, and those patients who are likely to receive little benefit from OART are treated with the current SOC. Correlation analysis was utilized to identify univariate reference plan predictors of adaptive benefit, the most promising of which was used for optimal patient selection. Finally, a multivariate logistic regression model was trained to holistically stratify high- and low-benefit OART APBI candidates. The difference in adaptive benefit between those identified as SOC and adaptive was tested for the univariate and multivariate models.

## 2. Methods

### 2.1. Patient cohort

Twenty-nine patients (31 targets because two patients received bilateral treatment) with early-stage breast cancer received online adaptive APBI treatment (30 Gy in five fractions) between January of 2022 and August of 2023 in this single-institutional, retrospective study, which is covered under an Institutional Review Board approved protocol (IRB-120703005). A comprehensive summary of patient age, laterality, target and breast volumes, gross tumor volume to clinical target volume (GTV-CTV) margins, CTV-PTV margins, tumor staging, fiducial placement, motion management, and treatment plan type is given in table 1. Patients were scanned using a Phillips Brilliance big bore CT scanner with 1 mm slices and were immobilized according to institutional protocol. GTVs were defined as the lumpectomy cavities, including pertinent surgical clips. CTVs were expanded from the physician-contoured GTVs via isotropic margins, and PTVs were expanded via isotropic CTV margins. CTVs and PTVs were cropped back from anatomical boundaries of disease spread (lungs, ribs, etc.) and skin (3 mm inward body expansion) for evaluation. Smaller CTV-PTV margins were utilized based on clinical staging and judgement by the radiation oncologist (i.e. lower-risk disease and/or a prohibitive PTV to breast ratio). Two targets were located inferiorly and posteriorly near the breast fold; due to increased anatomical uncertainty, the CTV-PTV margins were increased from 3 to 5 mm. Breath-hold motion management was utilized by the treating physician for select left-sided patients based on proximity of the target to the heart and the presence of prior RT; all other targets were treated free-breathing. Fiducials were utilized to aid in seroma delineation in the majority of targets (Coles *et al* 2009), as they have previously demonstrated a reduction in inter-observer target delineation variability (Lowrey *et al* 2021). However, they were omitted for targets with sufficient contrast between seroma and healthy tissue. IDENTIFY (Varian Medical Systems, Palo Alto, CA) surface monitoring enabled initial surface positioning and patient motion monitoring throughout treatment (Stanley *et al* 2023a).

### 2.2. Online adaptive treatment

#### 2.2.1. Machine and treatment planning system (TPS)

The Ethos (Varian Medical Systems, Palo Alto, CA) treatment unit is a kV-CBCT-guided, online-adaptive linear accelerator equipped with a 6 MV flattening filter free beam and dual stacked and staggered 10 mm MLC banks, enabling an effective MLC resolution of 5 mm. The dual banked MLCs act as jaws, with a maximum exposure area of 28 cm *×* 28 cm. The gantry rotates a maximum of four revolutions/minute and delivers a maximum dose rate of 800 MU/min, enabling faster treatments compared to traditional linear accelerators. The Ethos TPS is an online platform designed to generate both IMRT and volumetric modulated arc therapy (VMAT) plans, which are calculated using Acuros XB (AXB, v16.1.0) with dose-to-medium and a vendor-required 2.5 mm calculation grid.

#### 2.2.2. Online adaptive workflow

**Figure 1.**
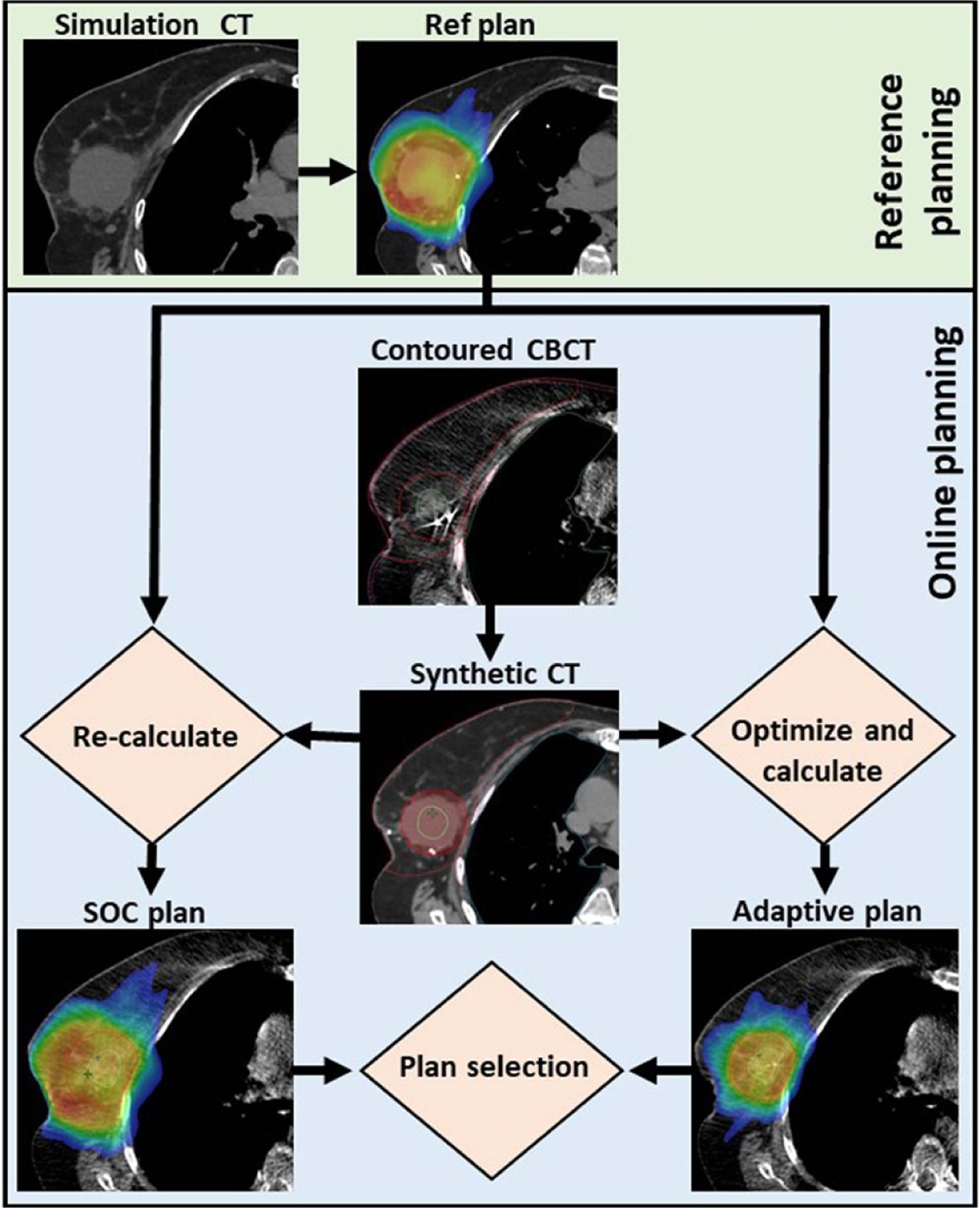
APBI treatment workflow using the Ethos kV-CBCT online adaptive treatment delivery system. A daily CBCT scan is automatically contoured, then edited as needed. A synthetic CT is generated by deformable image registration of the simulation CT to the planning CBCT via Velocity^™^. Ethos automatically generates a SOC and adaptive plan for every fraction by re-calculating the reference plan onto daily anatomy and optimizing a new plan using CBCT anatomy, respectively. The optimal plan was then selected by the treating physician. This process was repeated for each of five treatment fractions. Reproduced from Ghimire *et al* (2023).

While other OART institutional workflows has been previously published in detail (Stanley *et al* 2023b), our APBI process is summarized below and illustrated in figure 1. A reference plan was generated for each patient based on simulation CT images and structure sets, then optimized using planning objective templates submitted to the TPS. Our institution’s approach for reference planning entails minimizing dose to the heart due to the linear, non-threshold dose dependence of cardiac toxicity (Darby *et al* 2013, van den Bogaard *et al* 2017, Laugaard Lorenzen *et al* 2020), followed by minimizing other ipsilateral OAR doses while maximizing conformity (Pogue *et al* 2023a). For a given adaptive fraction, two therapists, a physicist, and a physician were required to participate in the entirety of treatment. First, a CBCT scan was acquired and influencers, or structures important for influencing image deformation, were automatically segmented via the onboard AI (both breasts, heart, both lungs). These structures were then edited as necessary by the qualified medical physicist and reviewed by the treating physician. The GTV and other non-influencer normal tissues were then generated via a structure-guided deformable image registration. These structures were also edited as necessary. A CTV and PTV were derived from the GTV using prescribed structure derivations.

Because CBCT image systems have not been calibrated for dose calculations, a synthetic CT was created by automatically deforming the simulation CT to daily CBCT anatomy via the Velocity^™^ algorithm (Zhen *et al* 2012, Gao *et al* 2021). Next, two plans were generated for every fraction: a SOC plan was obtained by performing a global rigid registration between the daily and simulation anatomy focused on the target alignment, then re-calculating the reference plan onto the daily synthetic CT, and a new adaptive plan was optimized using the synthetic CT and objective template utilized for reference plan generation. The physician and physicist then selected the superior plan of the SOC and adapted plans, referred to as the delivered (Del) plan throughout this work. Prior to treatment, a position verification CBCT was performed to verify, and correct, if necessary, patient alignment. Lastly, the Mobius3D-Adapt (Varian Medical Systems, Palo Alto, CA) secondary calculation algorithm was used to verify correct plan MUs, a 5%/3 mm global gamma value above 95% (using a 10% threshold), and less than 5% mean and D90% difference between algorithms for all target structures, according to department guidelines (Zhao *et al* 2023). Based on a previous study from our institution, the reference planning (from simulation to first treatment, including weekends) and online planning/delivery processes (from initial CBCT to end of beam delivery) required median times of 26 d and 31 min, respectively (Pogue *et al* 2023b).

### 2.3. Dose-volume metrics

This work analyzes a total of 333 treatment plans; each of 31 targets received one reference plan, five SOC plans, and five adaptive plans. Due to issues exporting DICOM RT files from the Ethos TPS to the Eclipse TPS (Varian Medical Systems, Palo Alto, CA), eight plans could not be analyzed in Eclipse. One patient was missing two SOC and two adaptive plans, and two patients were missing one SOC and one adapted plan. Results for all five fractions are presented for the remaining 28 patients. Based on work by Rahimi *et al* (2017, 2021), the following planning goals were utilized throughout this work to minimize both acute toxicities (breast pain, hyperpigmentation, paresthesia, radiation dermatitis, fatigue, fibrosis) and late toxicities (breast pain, rib fracture, chest wall pain, radiation dermatitis, breast infection, telangiectasias, hyperpigmentation, fatigue, fibrosis, fat necrosis, radiation pneumonitis): PTV V100% ⩾ 95%, Ipsilateral breast V30Gy ⩽ 20%, Ipsilateral breast V15Gy ⩽ 40%, heart V1.5Gy ⩽ 40%, ipsilateral lung V9Gy ⩽ 10%, Skin D0.01cc ⩽ 39.5 Gy, Rib D0.01cc ⩽ 43 Gy, RTOG conformity index (CI) ⩽ 1.30, and high-dose spillage ⩽ 15%,

(1) $$\text{RTOG CI}=\frac{\text{PIV}}{\text{TV}}$$

(2) $$\text{Spillage}\left(\%\right)=100\times \frac{{\text{PIV}}_{105\%}-{\text{TV}}_{105\%}}{\text{TV}}.$$

PIV and TV are the prescription isodose volume and treated volume (i.e. PTV volume), respectively. Subscripts specify the isodose volume analyzed, if different than 100%. All per-fraction plan doses are scaled to full-course (five fraction) dose; this is the default Ethos export format, allows intuitive plan quality evaluation according to the planning goals above, and is a commonly used approach for presenting per-fraction adaptive plans with Ethos (Henke *et al* 2019, Astrom *et al* 2022, Mao *et al* 2022, Montalvo *et al* 2023). Dose accumulation was not performed due to the uncertainties associated with deforming variable and shrinking targets.

A comparison of Ref, SOC, and Del (i.e. the plans selected for treatment) dose metrics and planning objective compliance has previously been published by our institution for a sequential 21-target cohort (Pogue *et al* 2023b). Because our intent was demonstrating net improvement with adaptive capabilities, and because the SOC plan was superior to the adaptive plan in some cases, Del plans were compared to SOC and Ref plans, as opposed to exclusively comparing adaptive plans. Individual and population differences between mean Del metrics and both Ref metrics and mean SOC metrics were investigated, as well as DVH comparison for select patients. To measure patient-specific dosimetric improvement with adaptive capabilities $\left(\Delta \right)$, the difference between mean SOC plan metrics $\left(\bar{\text{SOC}}\right)$ and mean delivered plan metrics $\left(\bar{\text{Del}}\right)$ was evaluated using the average values over five fractions, with positive values indicating improvement. For example, $\Delta \text{Breast V30Gy}\left(\%\right)=\bar{{\text{SOC}}_{\text{BreastV30Gy}}}-\bar{{\text{Del}}_{\text{BreastV30Gy}}}$. All Ref, SOC, and adaptive dose volume histogram (DVH) metrics were extracted via the Eclipse Scripting Application Programming Interface (version 16.1).

### 2.4. Patient classification

To stratify low- and high-yield OART APBI patients, median per-patient dosimetric benefits with adaption were calculated for the metrics listed in table 2. Patients were then classified as high-yield (i.e. optimal OART candidates) if at least 5/10 metrics were at or above the median adaptive benefit value, otherwise they were classified as low-yield.

### 2.5. Univariate correlation

Simulation structure set and reference plan metrics were correlated with dosimetric improvements to elucidate which reference plan parameters enable univariate prediction of dosimetric benefit with adaption. Table 2 lists the 22 reference plan predictors and 10 dosimetric improvements utilized for univariate correlation analysis. Thus, 220 correlations were performed using the Spearman correlation $\left(r\right)$, which applies the Pearson correlation coefficient to ordinal data ranks. This statistical metric quantifies the magnitude of monotonic relationships in non-parametric data. Cohen’s standard of effect size was used to elucidate correlation strength, and p values for the probability of finding the observed correlation or stronger if no correlation exists were calculated. All r and p values presented in this manuscript were calculated using the scipy.stats library (v1.6.2) in python.

The magnitude of Spearman correlations $\left(\left|r\right|\right)$ between reference plan metrics and dosimetric beneefit was used to identify optimal univariate predictors. Then, Youden’s index was used to identify the optimal patient stratification threshold by maximizing the difference between true positive rate (TPR) and false positive rate for the univariate model relative to ground truth (section 2.4) (Hajian-Tilaki 2013). Youden’s index is a single, commonly utilized measure of a model’s ability to balance sensitivity (TPR) and specificity (TNR).

### 2.6. Machine learning logistic regression

A multivariate logistic regression model was trained to predict whether targets were low or high-yield (section 2.4), based solely on reference plan and simulation structure set metrics. Model features were systematically selected in two steps for optimal performance. A preliminary list of features was first obtained by utilizing the Mann–Whitney U test to evaluate difference between targets treated entirely with adaption and those treated with at least one SOC fraction for all 22 reference plan metrics; recursive feature elimination then allowed for identification of key model features from reference plan metrics with greater differences.

A binary logistic regression model for calculating the sigmoidal probability P that a given target would be high-yield was then constructed.


(3) $$P=\frac{1}{1+e^{-\left(\beta_{0}+\beta_{1}X_{1}+\beta_{2}X_{2}+\ldots +\beta_{n}X_{n}\right)}}$$

β values represent the model’s learned weights and X values represent individual features for n key features. Leave-one-out cross-validation (LOOCV) was performed to prevent bias when training the model using the entire dataset. For LOOCV, the model was trained on 30 targets, and the remaining target was classified. This process was repeated 31 times such that each target was classified once without bias, and the model was evaluated using the 31 ‘left-out’ predictions. To investigate the effects of data idiosyncrasies and noise on the model, areas under the curve (AUC) of receiver operating characteristic (ROC) curves were analyzed for the entire training dataset (i.e. model trained using all 31 targets) and the LOOCV model. The regularization hyperparameter, C=1/λ, was tuned to maximize LOOCV model performance as determined by AUC of the ROC. When many features are used for a limited amount of data, the resulting model may tailor to idiosyncrasies in the training data which are not present in the validation data. To address this issue, *λ* applies a penalty to large coefficients of the training cost function, thereby preventing training data overfitting for improvement in validation cohort performance. The optimal LOOCV probability threshold P was then obtained via Youden’s index. Machine learning recursive feature selection and logistic regression modelling were performed using python’s Scikit-learn library (v0.24.1).

Lastly, a comparative ROC analysis was performed between the univariate and multivariate models. Furthermore, to investigate the univariate and multivariate models’ ability to stratify high- and low-benefit targets, and to elucidate expected model performance if implemented into a clinic, differences in dosimetric benefit between targets identified as low-yield (SOC) and high-yield (adaptive) by both models were tested. To obtain viable sample size and statistical power, per-fraction results are compared. If both data sets were normal (i.e. *p >* 0.05 using the Shapiro–Wilk test), the unpaired Welch’s *t*-test was utilized for difference testing. Otherwise, the Mann–Whitney U unpaired, non-parametric test was utilized, with *p*⩽ *0.05* deemed significant.

## 3. Results

### 3.1. Effect of adaption on plan quality

**Figure 2.**
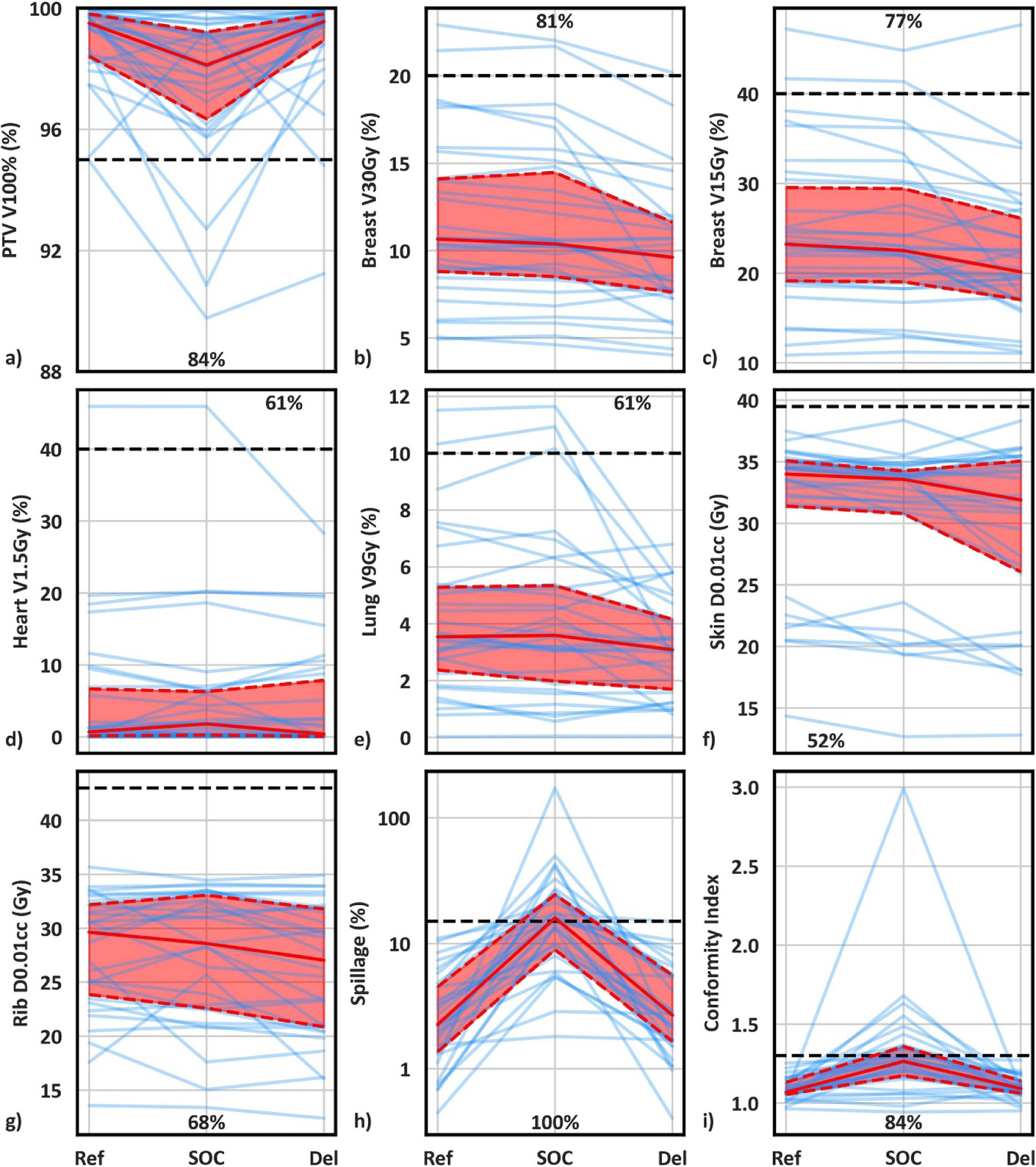
Line plots of reference, mean standard-of-care, and mean delivered plan metrics for individual patients (blue lines). Solid and dashed red lines illustrate the median and interquartile range, respectively, while horizontal black dashed lines show planning goals. Annotated values show the percentage of targets where mean delivered metrics were superior to mean standard-of-care metrics.

**Figure 3.**
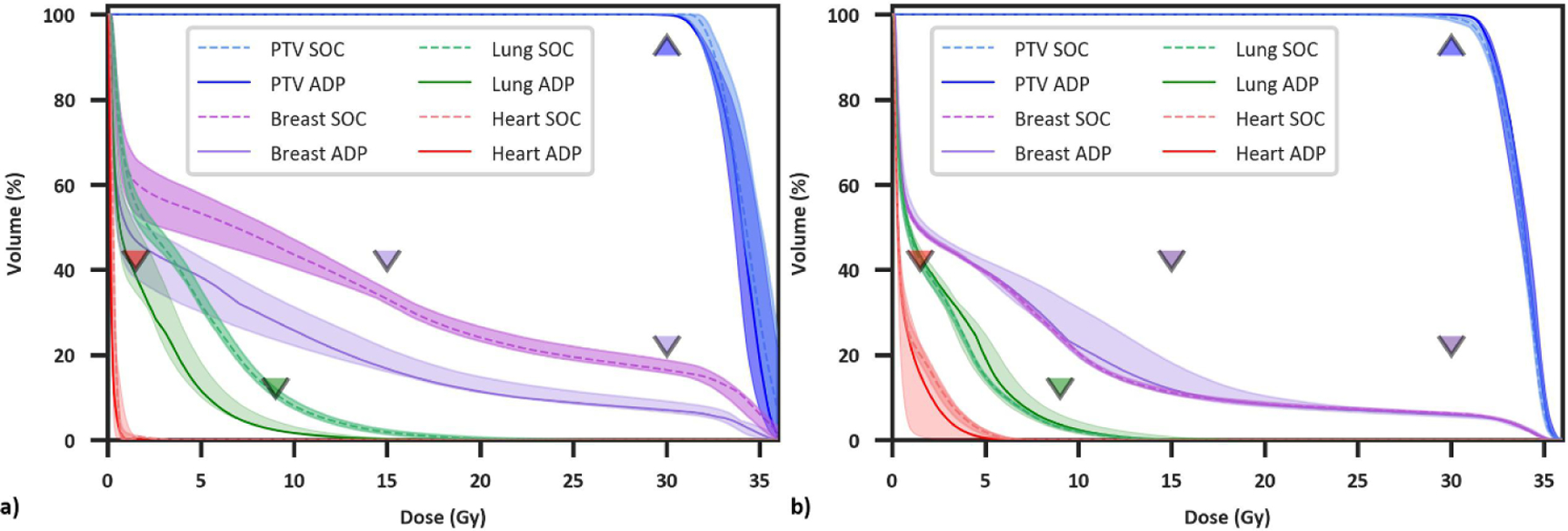
Dose volume histograms for two patients receiving significant (a) and negligible (b) benefit from OART, respectively. Dashed/solid lines and shaded regions illustrate the median and minima/maxima, respectively, for five standard of care and adaptive fractions, and triangle points represent planning objectives. The adaptive plan was selected five times for patient (a) and twice for patient (b).

Ref (initial plans based on simulation CT), mean SOC (ref plans recalculated onto daily anatomy), mean Del metrics (choice of either the SOC or daily adapted plan), and statistical comparisons are summarized in supplemental table S1, which are consistent with previous findings favoring OART for APBI (Pogue *et al* 2023b). Lineplots showing Ref, SOC, and Del plan metrics for individual patients, as well as median and interquartile range values, are shown in figure 2. Individual patient trends are noisy although population trends are very clear. More explicitly, although there is a net improvement in Del plans compared to SOC and Ref plans, with Del plans resulting in fewer planning objective violations, there is clear patient-specific variation in benefit with adaption. This is supported by the fact that mean SOC metrics were superior to mean Del metrics in at least 30% of patients for 4/9 metrics (figures 2(d)–(g): Heart V1.5Gy, Lung V9Gy, Skin D0.01cc, and Rib D0.01cc). For these four metrics, Del values were equal to or improved to SOC values for 70%, 69%, 60%, and 70% of individual fractions, respectively, indicating a slight but noticeable difference between trends in total fractions and fraction means. Differences in dose volume histograms between a patient treated with 5/5 adaptive fractions (figure 3(a)) and a patient treated with 2/5 adaptive fractions (figure 3(b)) further support significant variation in adaptive benefit. Figure 3(a) illustrates a dramatic drop in ipsilateral breast and lung dose with adaption whereas figure 3(b) shows negligible benefit with adaption, as SOC ipsilateral breast and lung dose were lower overall than adaptive lung dose.

### 3.2. Univariate analysis

**Figure 4.**
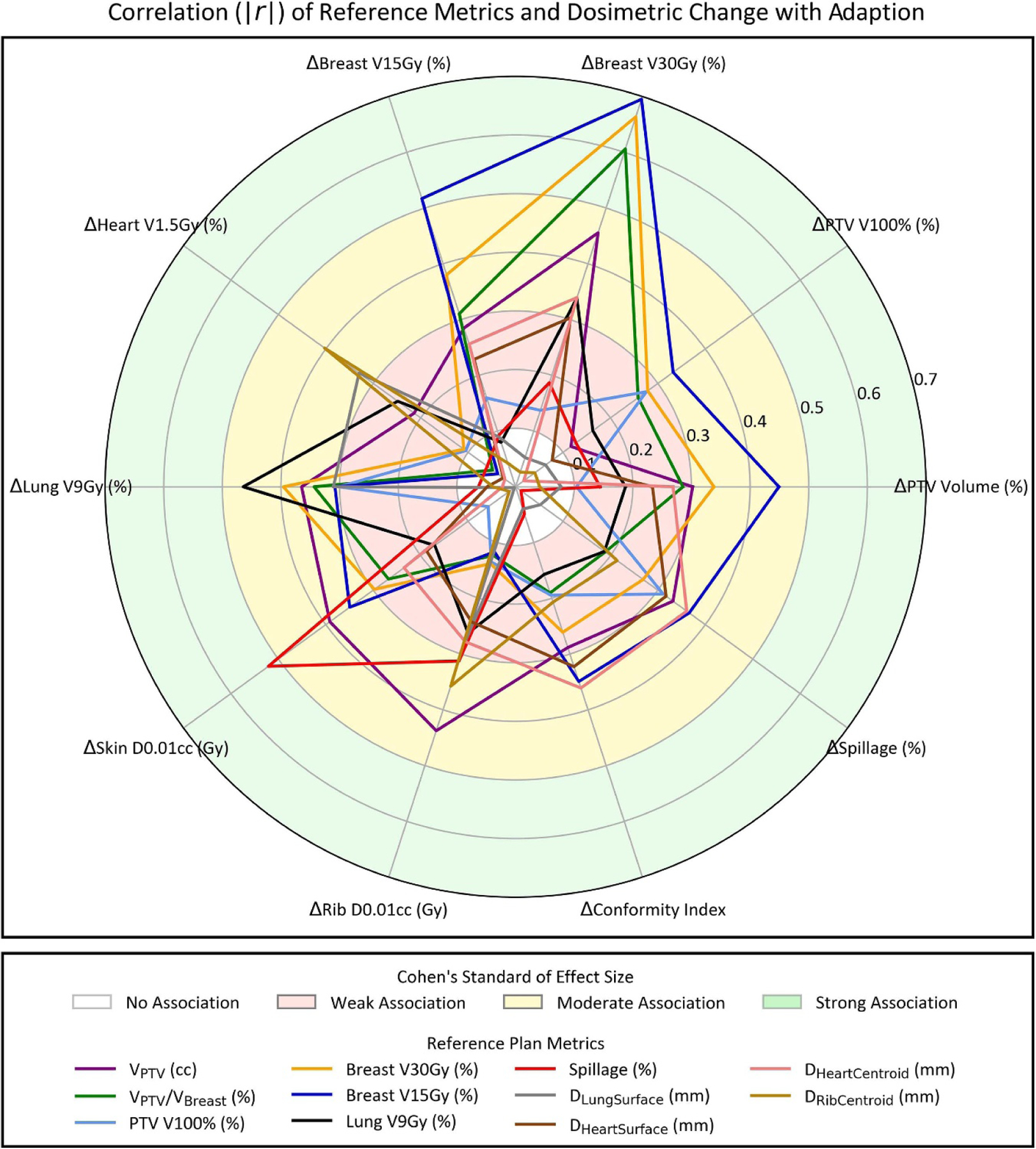
Radar plot showing the magnitude of Spearman correlations $\left(\left|r\right|\right)$ between reference plan metrics and dosimetric benefits with adaption (i.e. Delivered minus standard-of-care plan metrics). Reference metrics with two or more moderate to high correlations (r  ⩾  0.30) are shown. Shaded regions illustrate the strength of association according to Cohen’s standard of effect size.

Correlating all reference plan metrics and dosimetric benefits with adaption yielded 42 Spearman r magnitudes ⩾0.30, corresponding to moderate and large associations according to Cohen’s standard of effect size (supplementary table S2). Similarly, 19 p values from testing the null hypothesis of no correlation were ⩽0.05 (supplementary table S3). To clearly visualize the largest multivariate drivers of dosimetric benefit with adaption, a radar plot was generated for all reference plan metrics with at least two correlations of 0.30 or higher (figure 4). Eleven reference metrics resulted in two or more $r\;⩾\;.30:V_{\text{PTV}}\left(\text{cc}\right)$, *V*_PTV_/*V*_Breast_ (%), PTV V100%, ipsilateral breast V30Gy (%) and breast V15Gy (%), ipsilateral lung V9Gy (%), spillage (%), *D*_LungSuface_ (mm), *D*_HeartSuface_ (mm), *D*_HeartCentroid_ (mm), and *D*_RibCentroid_. Six reference metrics correlated moderately or highly with three or more dosimetric improvements. An interesting observation is that proximity of PTV and heart (surface and centroid) at least partially determined whether reference plan spillage and CI was tenable during adaptive treatment.

**Figure 5.**
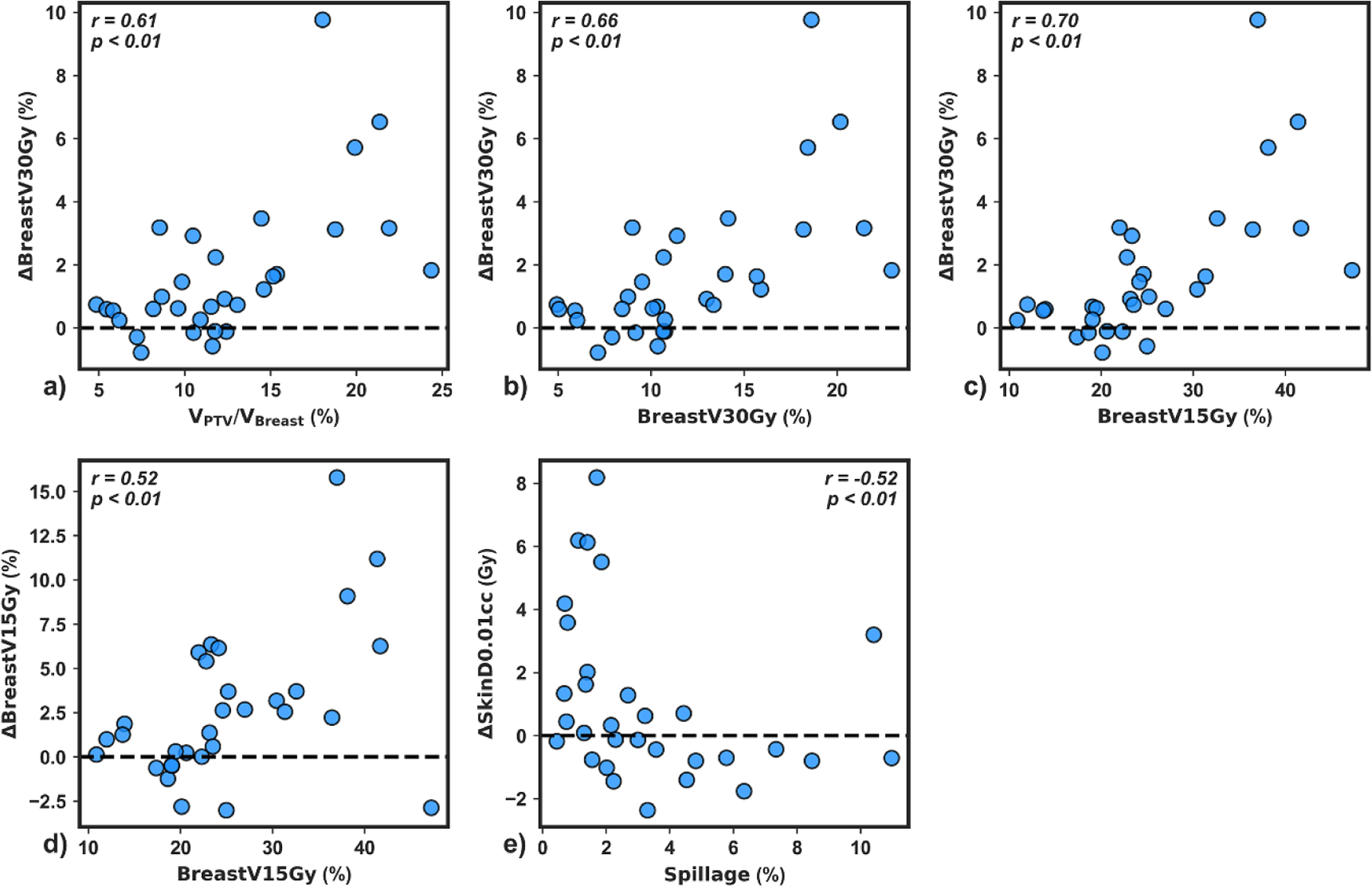
Mean dosimetric benefit versus reference plan metric for the five strong univariate correlations observed in this study (r  ⩾  0.50). Spearman correlation values and p-values testing the null hypothesis of no correlation are shown for each plot.

To elucidate the impact of individual planning metrics on univariate dosimetric improvement, scatterplots of dosimetric benefit versus reference metric were generated for the five metrics demonstrating strong correlations with indicators of dosimetric improvement (r  ⩾  0.50). *V*_PTV_/*V*_Breast_ (%), ipsilateral breast V30Gy (%), and ipsilateral breast V15Gy each had a strong positive association with $\Delta \text{Breast V30Gy}\left(\%\right)$ (figures 5(a)–(c), r  ⩾  0.61, *p <* 0.01). Ipsilateral breast V15Gy positively self-correlated (figure 5(d), r=0.52, *p* ⩽ 0.01). Interestingly, a reduction in Δskin D0.01cc was observed for spillage values above about 3% (figure 5(e), r=−0.52, *p <* 0.01) indicating that reference stereotactic plan quality was a primary driver of reduction in skin D0.01cc with adaption. Ipsilateral breast V15Gy accounted for 2/5 correlations with r ⩾  0.50 (figure 5) and resulted in the highest correlation for 5/10 adaptive benefits (figure 4). Therefore, this simulation metric was selected for univariate patient classification.

### 3.3. Multivariate analysis

Targets classified as high-yield (n=16) received greater adaptive benefit compared to those identified as low-yield (n=15); this is supported by supplementary figure S1, which illustrates a 1.0% PTV V100% coverage reduction for high-yield targets relative to low-yield targets, but significant improvements for 8/10 metrics (excluding Δheart  V1.5Gy). These classifications were treated as ground truth when training the model. Five reference metrics were identified as key machine learning logistic regression model features for predicting optimal adaptive patients: GI, CI, *D*_HeartSuface_ (mm), and *D*_LungCentroid_ (mm), and ipsilateral breast V15Gy (%). Positive model prediction (1) suggested a target would be recieve high-benefit, while negative model prediction (0) suggested a target would receive low adaptive benefit.

### 3.4. Model comparison

**Figure 6.**
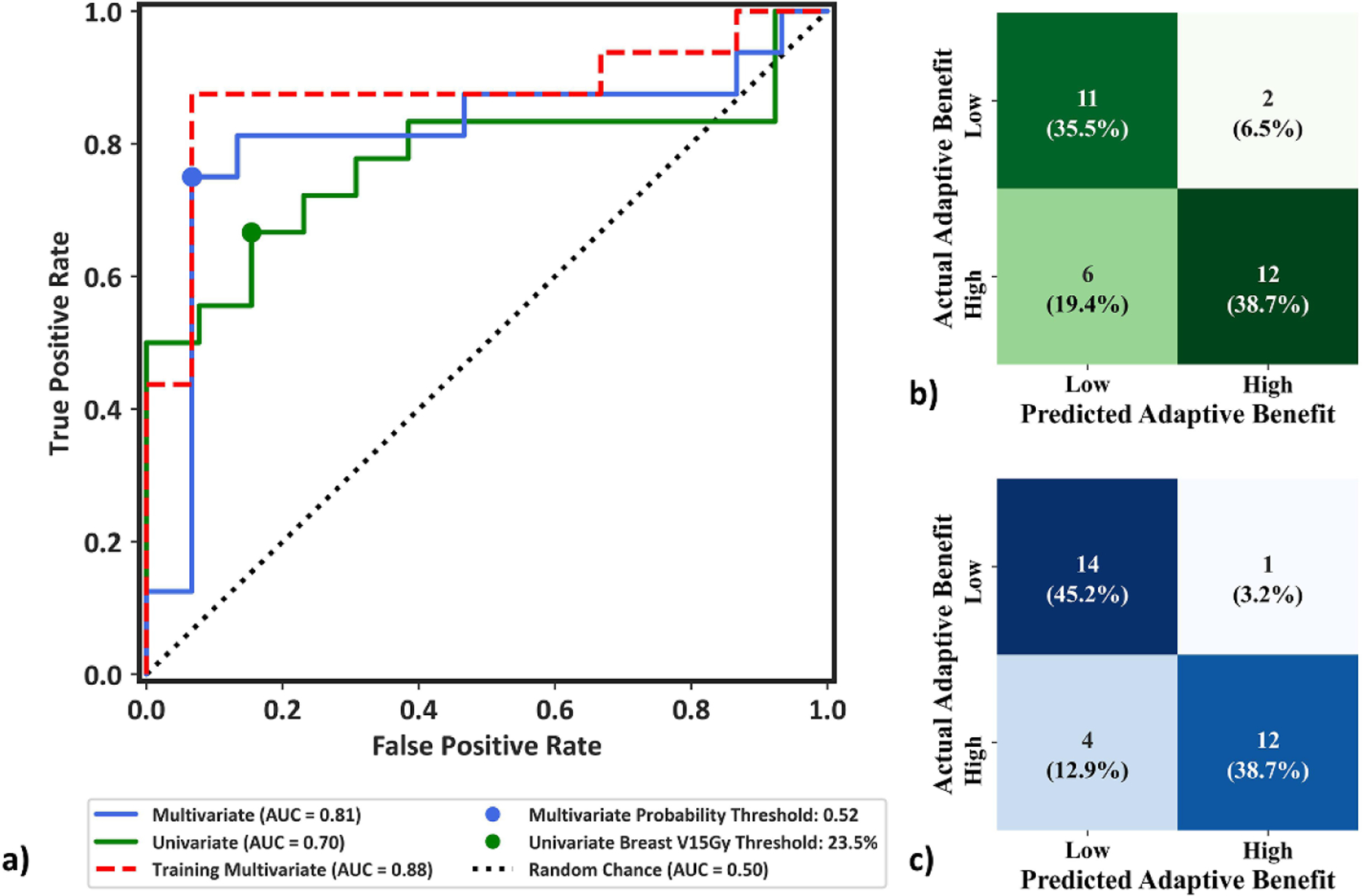
(a) ROC curve from using the univariate model, training the multivariate model with the entire dataset, and using the LOOCV multivariate model, illustrating the binary model performance at varying probability thresholds without bias towards class imbalance. The dashed line indicates the performance of a ‘random chance’ binary classification model as measured by area under the curve. Youden’s indices (circles) illustrate the thresholds resulting from maximum differences between true positive and FPR. (b) Confusion matrix heat map for the univariate ipsilateral Breast V15Gy model using a threshold of 23.5%. (c) Confusion matrix heat map of the multivariate validation model for optimal probability threshold P and regularization parameter C values of 0.52 and 0.006, respectively.

**Figure 7.**
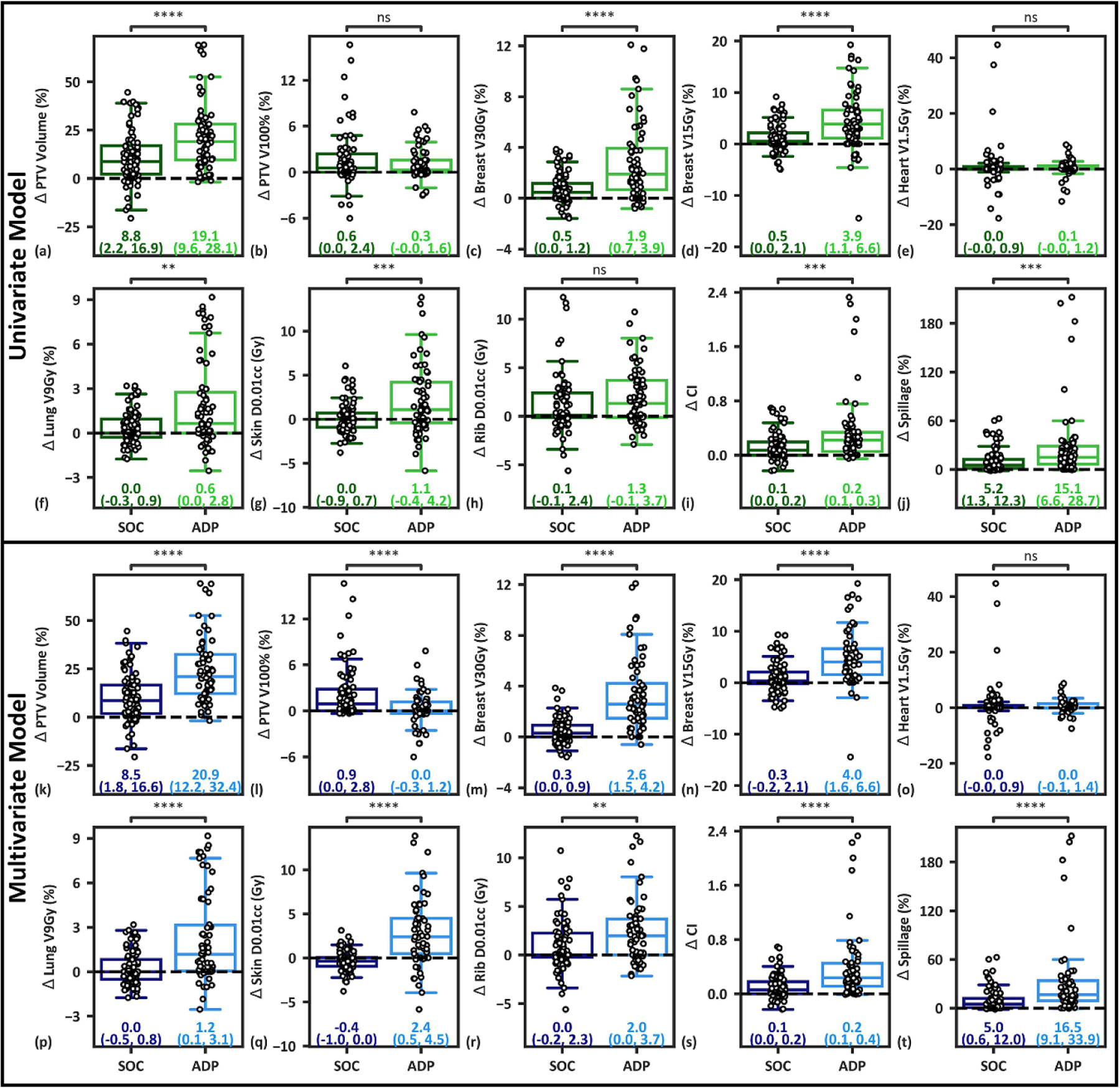
(a)–(j): Comparison of per-fraction plan metrics between 14 targets identified as high-yield (68 fractions, ADP) and 17 targets identified as low-yield (83 fractions, SOC) using the univariate model. (k)–(t): Comparison of per-fraction plan metrics between 13 targets (62 fractions) identified as high-benefit and 18 targets (89 fractions) identified as low-benefit by the leave-one-out cross validation multivariate model. Median and interquartile ranges are presented for each cohort. Positive values indicate improvement with adaption. If both data sets were normal (i.e. *p >* 0.05 using the Shapiro–Wilk test), the unpaired Welch’s *t*-test was utilized for difference testing. Otherwise, the Mann–Whitney U unpaired, non-parametric test was utilized. Significance values are stratified as follows: ns: *p >* 0.05; ^∗^: 0.01 *< p* ⩽ 0.05; ^∗∗^: 0.001 *< p* ⩽ 0.01; ^∗∗∗^: 0.0001 *< p* ⩽ 0.001; ∗∗∗∗: *p* ⩽ 0.0001.

The AUC of the univariate model, multivariate training dataset, and multivariate LOOCV model were 0.70, 0.88, and 0.81, respectively (figure 6(a)). For the univariate ipsilateral breast V15Gy model, Youden’s index determined an optimal breast V15Gy threshold of 23.5%, resulting in 14/31 targets being identified as high-yield and 74.2% accuracy (figure 7(b)), defined as total correct prediction. The optimal multivariate logistic regression model resulted in 13/31 targets being identified as high-yield and 83.9% model accuracy (figure 7(c)). This illustrates that while there is decrease in LOOCV model performance relative to the training dataset due to data idiosyncrasies, the LOOCV model still outperforms the univariate model. The consequences of negative classification were deemed clinically imperative, and investigated further. Four of the 31 targets utilized in this study had reference plans containing one or more failing target or OAR constraint, which all passed with adaption; these targets were identified as receiving critical adaptive benefit. However, only one of these targets was identified as negative (TN or FN) by the univariate and multivariate models. Thus, 3.2% (1/31) of targets identified in the proposed models would be treated using SOC when adaption offered critical benefit.

Targets identified as high-yield by the univariate model received significant improvements for all metrics besides the PTV V100%, heart V1.5Gy, and Rib D0.01cc relative to low-yield targets (figures 7(a)–(j)). The multivariate model resulted in a 0.9% reduction in in per-fraction PTV V100% coverage for high-yield targets, but significant improvements relative to low-yield targets for every other metric besides the heart V1.5Gy (figures 7(k)–(t)), suggesting the model is adept at bifurcating low- and high-benefit OART patients. The multivariate model had similar breast and plan quality improvements relative to the univariate model, but showed large improvements in lung, skin, and rib metrics and was closer to ground truth classifications (supplementary figure S1).

## 4. Discussion

This work demonstrated the feasibility of constructing univariate and multivariate models for classification of low- and high-yield OART patients based on reference plan metrics. First, our data suggests that while adaptively delivered APBI plans were superior to both reference and SOC plans for most plan metrics, individual patients received varying adaptive benefit (figures 2 and 3). We then report that a large number of reference plan metrics (n=42) have moderate to strong univariate correlations with metric-specific adaptive benefits (supplementary table S1), enabling prediction of select adaptive benefits based on plans using the simulation CT. Targets with reference ipsilateral breast V30Gy and V15Gy metrics greater than 10% and 25%, respectively, received greater breast sparing with OART. Additionally, reference plans with spillage values below about 2.5% received greater skin sparing with OART. Lastly, a univariate Breast V15Gy model and a multivariate validation model performed with 74.2% (AUC=.70) and 83.9% accuracy (AUC=0.81), respectively (figure 6). Each model resulted in statistically significant differences in adaptive benefit between targets identified as SOC and adaptive for several metrics (figure 7), but the multivariate model resulted in superior results and more closely reflected ground-truth classification. The proposed univariate and multivariate models would effectively enable a clinic to select which patients to treat using OART and the SOC for better resource allocation prior to the start of treatment.

The proposed multivariate model was trained to predict optimal candidates for OART prior to the start of treatment for two reasons. First, triggering adaption based on the difference between reference plan and treated plan metrics would require that at least 1/5 fractions are treated non-adaptively, and thus 20% of potential adaptive benefit is lost. Second, the Ethos system does not allow users to treat adaptively based on non-adaptive treatments in real time; this requires offline replanning in the TPS. It is estimated that the planning processes for switching from non-adaptive to adaptive treatments would require at least 2 d. Therefore, it is likely that 2/5 fractions would still be delivered non-adaptively if we implemented an adaptive ‘triggering’ workflow, such as the one proposed by Lezzi *et al* for 15 fraction whole breast treatment (Iezzi *et al* 2022). And although they exhibited slightly better model results, with an AUC=0.86 versus 0.81 using our LOOCV model, their proposed workflow would come at the cost of treating approximately 40% of fractions non-adaptively for high-yield patients. Thus, while there is slightly inferior performance of this predictive model to a similar model in the literature, it allows the treatment workflows to be selected a priori, which is considered paramount for five-fraction APBI.

Some of the univariate adaptive benefit correlations observed here are not surprising, and are even predictable due to self-correlation and collinearities. For example, greater *V*_PTV_ (cc), *V*_PTV_/*V*_Breast_, and ipsilateral breast V30Gy and V15Gy reference plan metrics correlated with greater Δbreast  V30Gy (figures 4 and 5), greater ipsilateral breast V15Gy correlated with greater Δbreast  V15Gy (figure 5(d)), and greater lungV9Gy correlated with greater Δlung  V9Gy (figure 4). These results strongly suggest that larger targets with higher OAR dose metrics will benefit more from OART than smaller targets with favorable OAR dose; this further suggests that with utilization of OART, APBI may be safely offered to larger target volumes than previously recommended (Rahimi *et al* 2017). As such, ideal candidates for OART may be patients with high OAR dose metrics that still meet initial OAR planning constraints. Future research may clarify whether the standard planning template is geared towards larger targets, and if editing the optimization objectives leads to greater adaptive benefit for small targets. Furthermore, several unexpected correlations were observed. Among the most interesting were that plan quality metrics appear to drive skin sparing with adaption (figures 4 and 5(e)), and proximity of heart and PTV surfaces and centroids appears to drive reduction in high-dose spillage with adaption (figure 4). With this information, the treating physician can focus on reference plan quality metrics if acute or long-term skin toxicity is of particular concern. Furthermore, the entire treating team can expect a certain level of high dose conformity, relative to the reference plan, based on proximity of the target and heart.

Ground-truth stratifications were performed by classifying patients with at least five dosimetric improvement metrics at or above the median population-wide value as high-yield. Each patient not correctly classified by the multivariate model (5/31) had between three and seven dosimetric improvement metrics at or above population-wide median values, suggesting that the model only misclassified patients near the classification threshold. This is supported by the fact that most of the difference in adaptive benefit between ground truth classifications (supplementary figure S1) is captured by the multivariate model (figures 7(a)–(j)). In contrast, three patients classified by the univariate model as non-adaptive had eight dosimetric improvement metrics at or above population-wide median values, suggesting that these patients would lose significant adaptive benefit if the model were utilized clinically. However, it is also important to note that the univariate model suggests classifying patients using a simple ipsilateral breast V15Gy threshold, allowing for simple implementation without the loss of direct interpretability inherent to the multivariate model.

It is critical to highlight that univariate and multivariate results presented here are largely possible because our institution follows a standardized, template-based planning workflow for this site (Pogue *et al* 2023a). While dosimetrists alter objective values and priorities on a patient-by-patient basis in order to meet planning constraints, the plans presented are largely derived from the same planning approach; this includes adaptive plans, since they are optimized with the same template used for reference plan generation. As our clinic heavily prioritizes cardiac sparing due to its linear, non-threshold dose dependence, proximity of heart and PTV surfaces drives Δspillage and ΔCI, and constitutes a key machine learning model feature. Similarly, consistency in optimization structures intended to improve conformity and gradients likely leads to the trends observed in figure 5(e). With the largely systematic planning approach used for developing the plans used to train the model, it is expected that the specific anatomic and dosimetric quantities selected by the model may differ if trained on datasets from an institution with different planning priorities. That being said, the general approach outlined in this study could be applied to multi-institutional datasets to develop a more general model.

In addition to trends in adaptive benefit tied to the structure of our optimization template, PTV volume shrinkage and deformation are considered primary drivers of adaptive benefit, as discussed and illustrated at length previously (Pogue *et al* 2023b). The median PTV shrinkage at adaption relative to simulation was 13.2% for this 31-target cohort. It follows that reduced volumes of tissue are irradiated when treating smaller PTVs, and thus OAR dose should also decrease. Furthermore, it can be seen in figures 7(a) and (k) that the proposed models largely predict which targets will shrink more at adaption. However, target deformation is also believed to play role in OAR dose reduction due to the fluidity of seroma GTVs and pendulous nature of breast targets. Figure 2(b) shows that prescription isodose volumes decrease from Ref to SOC, even though the median SOC PTV volume is 13.2% lower. This provides dosimetric evidence for target deformation, as a smaller target with identical shape should still be entirely covered during scheduled treatment.

Mean dosimetric benefits with adaption were evaluated throughout this work as patient- and metric-specific representations of OART benefit. However, benefits were not perfectly consistent across fractions, even for individual patients. Population median (interquartile range) adaptive benefit values are 1.1% (0.3%–2.1%) for PTV V100%, 0.9% (0.4%–2.6%) for breast V30Gy, 0.1% (*−*0.3%–1.4%) for lung V9Gy, 0.1 Gy (*−*0.7–1.8 Gy) for skin D0.01cc, and 11.0% (4.4%–18.8%) for high-dose spillage. Future research may highlight whether these variations are stochastic or follow a trend with delivered fraction.

The proposed models suggested negative binary model classification for a patient receiving critical adaptive benefit, defined as meeting all planning goals with adaption when one or more goals failed in the reference plan. If one assumes that the planning goals in this study are surrogates for tumor control and normal tissue complication, treating a patient with SOC whose reference plan fails to meet all objectives leads to increased risk of normal tissue complication and decreased likelihood of tumor control, as adaption may enable all goals to be met. A viable solution would be to only implement the proposed model if all target and OAR goals are met in the reference plan, mitigating the risk of missing critical adaptive benefits by exclusively bifurcating lower risk patients. Even still, the only way to guarantee that adaptive benefit is maximized is to treat all patients adaptively; however, this is already infeasible for some clinics due to cost, and will likely become more prohibitive in the future with further adoption of OART.

Limitations of this study include a smaller patient cohort and the exploratory nature of the multivariate model. For example, non-binary classification could have been utilized instead of classifying patients as high- or low-yield, potentially leading to increased ability to capture adaptive benefit. Furthermore, utilization of an alternative model (e.g. random forest) and features (e.g. age, breast dimensions, laterality, etc.) may also lead to improvement in predictive capabilities. As such, this work presents potential methods for a priori patient stratification, but may potentially be improved upon using alternative methods.

CBCT image quality is perhaps the largest limitation of using Ethos for APBI. For this reason, our institution utilizes fiducials to aid in seroma delineation and reduce inter-observer variability when excellent GTV to healthy tissue contrast is not present. However, CT and CBCT image artifacts are in turn caused by the fiducials themselves, creating three sources of uncertainty. First, daily synthetic CTs will be inaccurate if material HUs are not appropriately assigned to Titanium and water on the simulation CT structure set because synthetic CTs are deformed from the simulation CT. Second, the synthetic CT will be spatially inaccurate if daily image artifacts prohibit accurate contouring. Lastly, uncertainty in seroma delineation due to artifact leads to uncertainty in PTV volume, which is expanded from the GTV. The authors do not believe these effects to be large, but they require further investigation to quantify, particularly for the Ethos platform. Importantly, uncertainties are expected to be less than the typical CTV-PTV expansion utilized (3 mm), and are therefore accounted for, but at the expense of greater OAR dose which is especially relevant in the adaptive setting. Lastly, departmental procedures dictate the SOC plan be selected if target delineation is prohibitive, as assessed by the physician and physicist, which was not observed for this cohort.

Lastly, there are several Ethos-specific limitations that should be discussed: synthetic CT reliability, the treatment system’s inherent 2.5 mm calculation grid, and automated SOC alignment. A robust investigation of synthetic CT reliability is outside the scope of this study; however, several groups have validated Ethos synthetic CT generation. Average differences of 1.6% between measurements and synthetic CT calculations have been observed when simulating weight losses and gains of up to 4 cm, which are significantly greater than the anatomical changes observed in this study (Kisling *et al* 2022). Additionally, *<*2% disagreement between synthetic CT and CBCT images in high dose regions of palliative spine treatments was observed (Nelissen *et al* 2023). Although small calculation grids are ideal for SBRT plans, the Ethos platform (v1.1 MR3) currently offers a minimum dose calculation resolution of 2.5 mm. And although GTV seromas were sometimes quite small (minimum=1.1cc), PTV volumes were much larger due to GTV-PTV margin expansions of about 1.3 cm, corresponding to minimum and median volumes of 28.7 cc and 101.4 cc, respectively. To exclude small volume calculation uncertainty, only PTV coverage was evaluated in this work. Thus, while a smaller calculation grid size is preferred, 2.5 mm is compliant with professional guidelines (Benedict *et al* 2010) and is considered acceptable for these target sizes. Adaptive plans were generated to conform to daily anatomy, whereas SOC plans were aligned using a global registration of CBCT and CT targets. SOC plans could not be tuned manually prior to calculation, like in a traditional image-guided RT workflow, creating the potential for misalignment error. However, significant disagreement between prescription coverage, as visually discerned using the SOC dose wash, and targets has not been observed, and the authors argue this to be the smallest source of error associated with Ethos. In fact, the SOC target coverage was sometimes greater than that of the adaptive plan optimized to daily anatomy, which was one of the deciding factors for choosing a SOC plan over the adaptive in those cases.

It is critical to mention that many of the limitations discussed above will be largely mitigated with the addition of HyperSight and Ethos 2.0 (Robar *et al* 2023); image quality will drastically increase, reducing artifact and enabling direct dose calculation, thus removing synthetic CT uncertainty. Additionally, high-fidelity mode stereotactic planning will utilize a 2 mm calculation grid.

## 5. Conclusion

This retrospective exploratory study investigated the feasibility of utilizing univariate correlation analysis and multivariate machine learning for identification of optimal OART APBI candidates. The presented work suggests that a correlation exists between pre-treatment dosimetric factors that could be modeled to enable stratification of high- and low-benefit OART candidates. Developing such strategies for patient selection could save clinics cost and resources associated with OART. Future prospective studies are needed to validate these findings.

## Supplementary Material

Supplementary materials

## Figures and Tables

**Table 1.** Patient cohort and planning description.

	Median (Min, Q1–Q3, Max)
Number of targets	31

Number of patients	29
Age (years)	67 (50, 58–73.0, 79)
Laterality	Left: 12, Right:19
GTV volume (cc)	13.5 (2.4, 8.4–23.2, 68.7)
CTV volume (cc)	68.4 (17.1, 55.8–88.1, 194.0)
PTV volume (cc)	98.7 (28.7, 79.7–128.0, 243.4)
Ipsilateral breast volume (cc)	874.1 (355.5, 629.6–1237.6, 2045.8)

GTV-CTV margin	5 mm: 4
	7 mm: 2
	8 mm: 1
	10 mm: 24

CTV-PTV margin	3 mm: 29
	5 mm: 2

Tumor staging	Stage 0: TisN0: 4
	Stage 1: T1aN0: 4
	T1bN0: 15
	T1cN0: 8

Fiducials placed	Yes: 26
	No: 5

Motion management	Breath hold: 3
	None: 28

Treatment plan type	IMRT: 9
	VMAT: 22

**Table 2.** Summary of all reference plan metrics and dosimetric changes with adaption utilized to perform univariate correlations.

Reference plan metrics	
*V*_PTV_ (cc)	PTV volume
*V*_Breast_ (cc)	Breast volume
*V*_PTV_/*V*_Breast_ (%)	Ratio of PTV to breast volume
*V*_Lung_ (cc)	Lung volume
PTV V100% (%)	Percent of PTV receiving prescription dose
Breast V30Gy (%)	Percent of breast receiving prescription dose
Breast V15Gy (%)	Percent of breast receiving 15 Gy (50% isodose volume)
Heart V1.5Gy (%)	Percent of breast receiving 1.5 Gy (5% isodose volume)
Lung V9Gy (%)	Percent of lung receiving 9 Gy (30% isodose volume)
Skin D0.01cc (Gy)	Maximum dose to 0.1cc of skin
Rib D0.01cc (Gy)	Maximum dose to 0.1cc of rib
RTOG CI	PIV/TV
Spillage (%)	100*×*(PIV_105_*−*TV_105_)/TV
Paddick GI	PIV_50_/PIV
*D*_LungCentroid_ (mm)	Distance between lung and PTV centroids
*D*_LungSurface_ (mm)	Distance between nearest lung and PTV surfaces
*D*_HeartCentroid_ (mm)	Distance between heart and PTV centroids
*D*_HeartSurface_ (mm)	Distance between nearest heart and PTV surfaces
*D*_RibCentroid_ (mm)	Distance between rib and PTV centroids
*D*_RibSurface_ (mm)	Distance between nearest rib and PTV surfaces
*D*_SkinCentroid_ (mm)	Distance between skin and PTV centroids
Time_SimToTreat_ (days)	Time from simulation to first adaptive fraction
Dosimetric change with adaption	

$\Delta \text{PTV volume}\left(\text{cc}\right)$	${\text{Ref}}_{\text{PTV volume}}-\bar{{\text{Del}}_{\text{PTV volume}}}$
$\Delta \text{PTV V100\%}\left(\%\right)$	$\bar{{\text{Del}}_{\text{PTV V100\%}}}-\bar{{\text{SOC}}_{\text{PTV V100\%}}}$
$\Delta \text{Breast V30Gy}\left(\text{\%}\right)$	$\bar{{\text{SOC}}_{\text{BreastV30Gy}}}-\bar{{\text{Del}}_{\text{BreastV30Gy}}}$
$\Delta \text{Breast V15Gy}\left(\text{\%}\right)$	$\bar{{\text{SOC}}_{\text{BreastV15Gy}}}-\bar{{\text{Del}}_{\text{BreastV15Gy}}}$
$\Delta \text{Heart V1.5Gy}\left(\text{\%}\right)$	$\bar{{\text{SOC}}_{\text{HeartV15Gy}}}-\bar{{\text{Del}}_{\text{HeartV15Gy}}}$
$\Delta \text{Lung V9Gy}\left(\text{\%}\right)$	$\bar{{\text{SOC}}_{\text{LungV9Gy}}}-\bar{{\text{Del}}_{\text{LungV9Gy}}}$
$\Delta \text{Skin D0.01cc}\left(\text{Gy}\right)$	$\bar{{\text{SOC}}_{\text{Skin}D0.01cc}}-\bar{{\text{Del}}_{\text{Skin}D0.01cc}}$
$\Delta \text{Rib D0.01cc}\left(\text{Gy}\right)$	$\bar{{\text{SOC}}_{\text{Rib}D0.01cc}}-\bar{{\text{Del}}_{\text{Rib}D0.01cc}}$
ΔCI	$\bar{{\text{SOC}}_{\text{CI}}}-\bar{{\text{Del}}_{\text{CI}}}$
$\Delta \text{Spillage}\left(\%\right)$	$\bar{{\text{SOC}}_{\text{Spillage}}}-\bar{{\text{Del}}_{\text{Spillage}}}$

PTV: planning target volume.

PIV: prescription isodose volume, body volume receiving specified dose.

TV: treated volume, target volume receiving specified dose.

## Data Availability

The data cannot be made publicly available upon publication because they contain sensitive personal information. The data that support the findings of this study are available upon reasonable request from the authors.
