# Supplementary material for: Unlocking the adaptive advantage: correlation and machine learning classification to identify optimal online adaptive stereotactic partial breast candidates
Source: Phys Med Biol. Author manuscript; Available in PMC 2024 Sep 19. (PMC11412112; doi:10.1088/1361-6560/ad4a1c)
Supplement: Supplementary materials [file NIHMS2015715-supplement-Supplementary_materials.docx]

**Supplementary Table S1.** Reference, mean standard-of-care, and mean delivered plan metrics for the 31 patient cohort utilized in this study. If both data sets were normal (i.e., p>0.05 using the Shapiro-Wilk test), the paired t-test was utilized for difference testing. Otherwise, the Wilcoxon paired, non-parametric test was utilized, with signficant p-values (<0.05) bolded and italicized.

|  | **Median (Q1-Q3): Ref plan and mean SOC and Treat plans for 32 patients** | | | | |
| --- | --- | --- | --- | --- | --- |
| **Metric** | **Ref** | **SOC** | **Del** | ***p*_Ref_Del_** | ***p*_SOC_Del_** |
| **PTV V100% (%)** | 99.5 (98.4-99.8) | 98.1 (96.3-99.2) | 99.6 (98.9-99.8) | 0.75 | ***<0.01*** |
| **Breast V30Gy (%)** | 10.7 (8.8-14.1) | 10.4 (8.5-14.5) | 9.6 (7.6-11.6) | ***<0.01*** | ***<0.01*** |
| **Breast V15Gy (%)** | 23.2 (19.1-29.5) | 22.5 (19.0-29.4) | 20.1 (17.1-26.1) | ***<0.01*** | ***<0.01*** |
| **Heart V1.5Gy (%)** | 0.7 (0.1-6.6) | 1.8 (0.2-6.3) | 0.4 (0.1-7.8) | 0.54 | 0.20 |
| **Lung V9Gy (%)** | 3.5 (2.4-5.3) | 3.6 (2.0-5.3) | 3.1 (1.7-4.2) | ***<0.01*** | ***0.03*** |
| **Skin D0.01cc (Gy)** | 34.0 (31.4-35.1) | 33.6 (30.8-34.3) | 31.9 (26.1-35.0) | ***<0.01*** | 0.36 |
| **Rib D0.01cc (Gy)** | 29.6 (23.9-32.2) | 28.6 (22.6-33.1) | 27.0 (20.9-31.8) | ***<0.01*** | ***0.01*** |
| **Conformity Index** | 1.07 (1.06-1.13) | 1.27 (1.17-1.36) | 1.09 (1.06-1.14) | 0.11 | ***<0.01*** |
| **Spillage (%)** | 2.3 (1.4-4.5) | 15.9 (8.9-24.5) | 2.7 (1.7-5.6) | 0.06 | ***<0.01*** |

**Supplementary Table S2.** Univariate Spearman correlation coefficients (*r*) of dosimetric change with adaption (columns) and simulation/reference plan metrics (rows). Magnitudes ≥ 0.30, corresponding to a moderate or greater association according to Cohen’s standard of effect size, are bolded and italicized. 42/220 correlations are ≥ 0.30.

|  | **ΔPTV Volume (cc)** | **ΔPTV V100% (%)** | **ΔBreast V30Gy (%)** | **ΔBreast V15Gy (%)** | **ΔHeart V1.5Gy (%)** | **ΔLung V9Gy (%)** | **ΔSkin D0.01cc (Gy)** | **ΔRib D0.01cc (Gy)** | **ΔCI** | **ΔSpillage (%)** |
| --- | --- | --- | --- | --- | --- | --- | --- | --- | --- | --- |
| **V_PTV (cc)** | **0.30** | -0.12 | **0.46** | 0.28 | -0.21 | **0.36** | **0.39** | **0.44** | 0.29 | **0.33** |
| **V_Breast (cc)** | 0.15 | -0.07 | 0.03 | 0.15 | -0.16 | 0.08 | 0.27 | 0.28 | 0.25 | 0.26 |
| **V_PTV/V_Breast (%)** | 0.29 | -0.26 | **0.61** | **0.31** | -0.05 | **0.34** | 0.27 | 0.12 | 0.19 | 0.19 |
| **V_Lung (cc)** | -0.14 | 0.12 | -0.11 | -0.16 | 0.21 | -0.08 | 0.00 | -0.06 | -0.08 | -0.08 |
| **PTV V100% (%)** | 0.10 | -0.28 | 0.14 | 0.16 | 0.10 | **0.30** | 0.06 | 0.13 | 0.19 | **0.31** |
| **Breast V30Gy (%)** | **0.34** | -0.28 | **0.66** | **0.38** | -0.11 | **0.40** | 0.30 | 0.14 | 0.26 | 0.27 |
| **Breast V15Gy (%)** | **0.45** | **-0.33** | **0.70** | **0.52** | -0.04 | **0.31** | **0.35** | 0.12 | **0.35** | **0.37** |
| **Heart V1.5Gy (%)** | -0.22 | 0.21 | -0.18 | -0.18 | 0.00 | -0.11 | 0.08 | 0.07 | -0.09 | -0.11 |
| **Lung V9Gy (%)** | 0.19 | -0.16 | **0.34** | 0.08 | 0.25 | **0.46** | 0.17 | 0.26 | 0.16 | 0.19 |
| **Skin D0.01cc (Gy)** | 0.07 | -0.17 | 0.17 | 0.13 | -0.12 | 0.20 | -0.27 | 0.11 | 0.12 | 0.14 |
| **Rib D0.01cc (Gy)** | 0.26 | 0.00 | 0.22 | 0.19 | **0.34** | -0.08 | 0.23 | -0.20 | 0.08 | 0.05 |
| **Conformity Index** | -0.03 | -0.24 | 0.01 | 0.05 | 0.16 | 0.01 | -0.24 | -0.12 | 0.06 | 0.12 |
| **Gradient Index** | -0.14 | -0.13 | -0.19 | -0.09 | 0.06 | -0.06 | -**0.52** | **-0.31** | -0.05 | -0.01 |
| **Spillage (%)** | -0.11 | -0.03 | -0.25 | -0.01 | 0.23 | **-0.30** | -0.22 | -0.22 | 0.00 | 0.03 |
| **D_LungSurface (mm)** | -0.08 | 0.06 | -0.05 | -0.09 | **-0.33** | **0.31** | 0.00 | 0.28 | 0.04 | 0.05 |
| **D_HeartSurface (mm)** | 0.23 | -0.08 | **0.30** | 0.23 | 0.03 | 0.05 | 0.19 | 0.24 | **0.32** | **0.32** |
| **D_RibSurface (mm)** | -0.20 | -0.05 | -0.17 | -0.14 | **-0.38** | 0.00 | -0.16 | 0.04 | -0.09 | -0.10 |
| **D_LungCentroid (mm)** | 0.11 | 0.10 | 0.12 | -0.05 | -0.05 | 0.20 | -0.12 | 0.00 | 0.04 | 0.03 |
| **D_HeartCentroid (mm)** | 0.27 | -0.02 | **0.34** | 0.26 | -0.02 | 0.02 | 0.24 | 0.28 | **0.36** | **0.36** |
| **D_SkinCentroid (mm)** | 0.13 | -0.02 | 0.20 | 0.19 | -0.24 | 0.11 | **0.35** | 0.25 | 0.14 | 0.06 |
| **D_RibCentroid (mm)** | 0.04 | 0.04 | -0.03 | 0.04 | **-0.40** | 0.04 | -0.01 | **0.36** | 0.21 | 0.21 |
| **Time_SimtoTreat (days)** | 0.29 | 0.14 | 0.23 | 0.24 | 0.07 | -0.18 | **0.31** | -0.05 | 0.11 | 0.08 |

**Supplementary Table S3.** P-values from testing the null hypothesis of no univariate, non-parametric correlation between dosimetric change with adaption (columns) and simulation/reference plan metrics (rows). P-values ≤ 0.05 are considered significant, and are bolded and italicized. 19/220 p-values are ≤ 0.05.

|  | **ΔPTV Volume (cc)** | **ΔPTV V100% (%)** | **ΔBreast V30Gy (%)** | **ΔBreast V15Gy (%)** | **ΔHeart V1.5Gy (%)** | **ΔLung V9Gy (%)** | **ΔSkin D0.01cc (Gy)** | **ΔRib D0.01cc (Gy)** | **ΔCI** | **ΔSpillage (%)** |
| --- | --- | --- | --- | --- | --- | --- | --- | --- | --- | --- |
| **V_PTV (cc)** | 0.10 | 0.53 | **0.01** | 0.12 | 0.25 | **0.04** | **0.03** | **0.01** | 0.12 | 0.07 |
| **V_Breast (cc)** | 0.41 | 0.73 | 0.88 | 0.42 | 0.38 | 0.68 | 0.13 | 0.13 | 0.17 | 0.16 |
| **V_PTV/V_Breast (%)** | 0.12 | 0.16 | **<0.01** | 0.09 | 0.80 | 0.06 | 0.15 | 0.51 | 0.31 | 0.31 |
| **V_Lung (cc)** | 0.46 | 0.53 | 0.55 | 0.40 | 0.25 | 0.66 | 0.99 | 0.73 | 0.65 | 0.67 |
| **PTV V100% (%)** | 0.58 | 0.13 | 0.46 | 0.39 | 0.57 | 0.10 | 0.76 | 0.47 | 0.29 | 0.09 |
| **Breast V30Gy (%)** | 0.06 | 0.13 | **<0.01** | **0.03** | 0.56 | **0.03** | 0.10 | 0.46 | 0.16 | 0.14 |
| **Breast V15Gy (%)** | **0.01** | 0.07 | **<0.01** | **<0.01** | 0.84 | 0.09 | 0.05 | 0.53 | 0.05 | **0.04** |
| **Heart V1.5Gy (%)** | 0.24 | 0.25 | 0.34 | 0.34 | 0.99 | 0.56 | 0.68 | 0.73 | 0.62 | 0.56 |
| **Lung V9Gy (%)** | 0.31 | 0.38 | 0.06 | 0.67 | 0.18 | **0.01** | 0.36 | 0.15 | 0.40 | 0.31 |
| **Skin D0.01cc (Gy)** | 0.70 | 0.36 | 0.35 | 0.47 | 0.52 | 0.27 | 0.14 | 0.54 | 0.53 | 0.46 |
| **Rib D0.01cc (Gy)** | 0.16 | 0.98 | 0.24 | 0.31 | 0.06 | 0.66 | 0.21 | 0.27 | 0.68 | 0.79 |
| **Conformity Index** | 0.89 | 0.20 | 0.95 | 0.78 | 0.38 | 0.96 | 0.19 | 0.53 | 0.77 | 0.53 |
| **Spillage (%)** | 0.44 | 0.49 | 0.31 | 0.62 | 0.75 | 0.74 | **<0.01** | 0.09 | 0.79 | 0.95 |
| **Gradient Index** | 0.54 | 0.85 | 0.18 | 0.97 | 0.21 | 0.10 | 0.24 | 0.24 | 0.99 | 0.87 |
| **D_LungSurface (mm)** | 0.68 | 0.74 | 0.78 | 0.63 | 0.07 | 0.09 | 0.99 | 0.13 | 0.83 | 0.78 |
| **D_HeartSurface (mm)** | 0.21 | 0.68 | 0.10 | 0.22 | 0.89 | 0.80 | 0.31 | 0.19 | 0.08 | 0.08 |
| **D_RibSurface (mm)** | 0.27 | 0.80 | 0.37 | 0.46 | **0.03** | 0.99 | 0.40 | 0.82 | 0.62 | 0.60 |
| **D_LungCentroid (mm)** | 0.56 | 0.60 | 0.52 | 0.80 | 0.79 | 0.27 | 0.53 | 1.00 | 0.81 | 0.87 |
| **D_HeartCentroid (mm)** | 0.14 | 0.93 | 0.06 | 0.16 | 0.90 | 0.91 | 0.20 | 0.13 | **0.05** | **0.05** |
| **D_SkinCentroid (mm)** | 0.47 | 0.90 | 0.28 | 0.29 | 0.19 | 0.57 | 0.05 | 0.18 | 0.46 | 0.74 |
| **D_RibCentroid (mm)** | 0.83 | 0.83 | 0.89 | 0.82 | **0.02** | 0.84 | 0.94 | **0.05** | 0.26 | 0.25 |
| **Time_SimtoTreat (days)** | 0.12 | 0.46 | 0.20 | 0.19 | 0.72 | 0.34 | 0.09 | 0.78 | 0.55 | 0.67 |


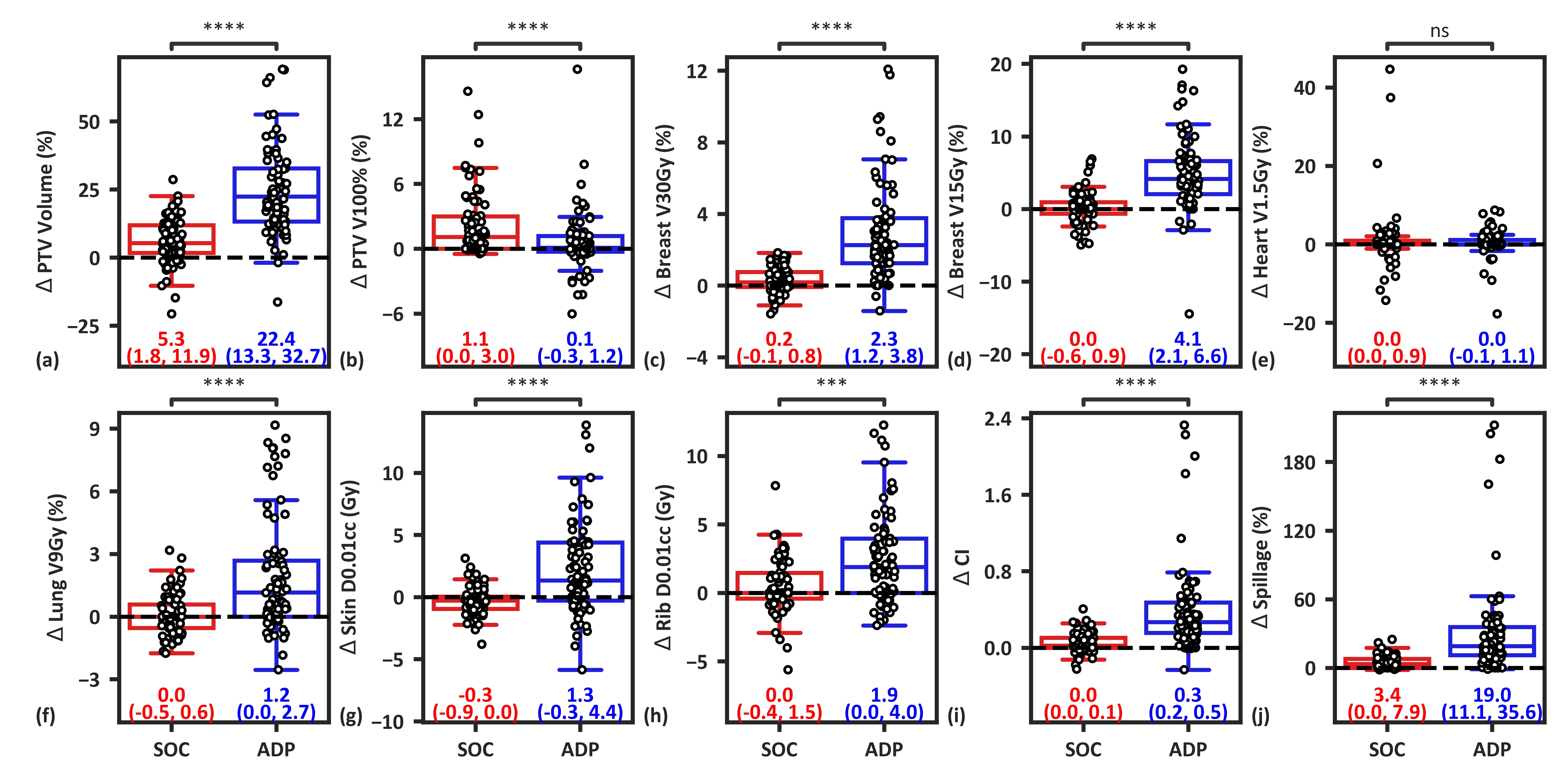


**Supplementary Figure S1.** Comparison of per fraction plan metrics between 16 targets identified as high-yield (77 fractions) and 15 targets identified as low-yield (74 fractions). Positive values indicate improvement with adaption. If both data sets were normal (i.e., p>0.05 using the Shapiro-Wilk test), the unpaired Welch’s t-test was utilized for difference testing. Otherwise, the Mann-Whitney U unpaired, non-parametric test was utilized. Significance values are stratified as follows: ns: *p* > 0.05; *: 0.01 < *p* ≤ 0.05; **: 0.001 < *p* ≤ 0.01; ***: 0.0001 < *p* ≤ 0.001; ****: *p* ≤ 0.0001.
